# Thyroid Hormone Signalling in Human Evolution and Disease: A Novel Hypothesis

**DOI:** 10.3390/jcm11010043

**Published:** 2021-12-23

**Authors:** Polyxeni Mantzouratou, Angelo Michele Lavecchia, Christodoulos Xinaris

**Affiliations:** 1 Department of Molecular Medicine, Istituto di Ricerche Farmacologiche Mario Negri IRCCS, Centro Anna Maria Astori, Science and Technology Park Kilometro Rosso, Via Stezzano 87, 24126 Bergamo, Italy; polyxeni.mantzouratou@marionegri.it (P.M.); angelomichele.lavecchia@marionegri.it (A.M.L.); 2 University of Nicosia Medical School, 93 Agiou Nikolaou Street, Nicosia 2408, Cyprus

**Keywords:** thyroid hormone signalling, human disease, human evolution, non-thyroidal illness syndrome, iodine, iodotyrosine, thyroid

## Abstract

Thyroid hormone (TH) signalling is a universally conserved pathway with pleiotropic actions that is able to control the development, metabolism, and homeostasis of organisms. Using evidence from paleoecology/palaeoanthropology and data from the physiology of modern humans, we try to assess the natural history of TH signalling and its role in human evolution. Our net thesis is that TH signalling has likely played a critical role in human evolution by facilitating the adaptive responses of early hominids to unprecedently challenging and continuously changing environments. These ancient roles have been conserved in modern humans, in whom TH signalling still responds to and regulates adaptations to present-day environmental and pathophysiological stresses, thus making it a promising therapeutic target.

## 1. The Evolution of TH Signalling as a Pleiotropic Effector

The basic elements for the emergence and evolution of life were made available by distinct astronomical events such as mergers of binary systems and core-collapse supernova [1,2] that took place billions of years ago. One of these basic elements, iodine, provided the key material for the evolution of thyroid hormone (TH) signalling—a universal and crucial molecular pathway for the evolution of life on earth.

The lives of the first unicellular organisms 3.5 billion years ago were crucially dependent on the availability of iodine, as it appears to have acted as a powerful antioxidant [3].

Iodine is one of the most electron-rich atoms that is available in the diets of marine and terrestrial organisms, and through peroxidase enzymes, its anion (iodide) could have served as a primitive electron donor in order to bestow antioxidant and catalytic functions on primitive iodide-concentrating cells [3]. Starting with these ancestral antioxidant and catalyst roles, iodine then coupled with tyrosine to form a more versatile and highly reactive molecule, iodotyrosine, which eventually formed iodothyronines through subsequent coupling reactions.

Organisms that cannot produce iodothyronines must acquire them from their food reference [4,5,6], which makes these organisms highly dependent on alimentary iodine. The dependence on iodothyronines and environmental iodide may have pressed for the selection of organisms that were able to first sense the availability of environmental iodo-compounds, and second undergo morphological/anatomical changes that would increase their ability to exploit environmental resources, as happens with echinoid larvae metamorphosis (for a detailed analysis of this hypothesis and supporting bibliography see Mourouzis et al [7]). In this way, iodothyronines were gradually converted from a sensing molecule into a strong regulator of both metabolism and development. Once iodothyronines had become key modulators of fundamental biological processes, organisms that were able to self-synthesise TH had gained a fitness advantage.

Due to these crucial roles in organisms’ adaptation to environmental conditions is not surprising that, over time, natural selection favoured the evolution of a sophisticated TH signalling that could sense and transmit environmental stimuli to cellular/genetic machinery (energy production, gene regulation and DNA replication in mitochondria), and ultimately orchestrate simultaneously metabolic and developmental processes. The coordinated and differential actions of TH signalling are mediated by the binding of the active form of TH (3,3,5-triiodo-l-thyronine, T3) to its receptors—the thyroid hormone receptors alpha and beta (TRα1–3 and TRβ1–3)—through which it regulates downstream signalling pathways and transcription factors [8]. Thyroid hormone receptors have separate expression patterns and divergent functional roles during foetal and adult life that are dependent on their liganded states [7,9]. TRα1 is the predominant isoform of TRs in many developing organs, while TRβ1 is widely expressed during adulthood. During foetal life, when T3 levels are low, TRα1 is highly expressed and acts as an apo-receptor (unliganded state) to repress adult genes and activate foetal ones. After birth, when T3 levels increase, the TRα1 switches to the holoreceptor form (liganded state) to induce the expression of adult genes, thus promoting cell maturation and physiological growth. In adulthood, circulating levels of THs are strictly regulated by the hypothalamic–pituitary–thyroid axis, which acts through a fine multi-loop feedback system, ensuring thyroid homeostasis. In addition, the availability of THs to tissue is locally regulated by the action of deiodinases (DIOs, DIO1, DIO2, DIO3). These selenocysteine-containing enzymes—the spatiotemporal expression of which changes during organ development in mammals—are capable of removing iodide from iodothyronines, turning T4 into T3 (DIO1 and DIO2) or catalysing the inactivation of T4 and T3 (DIO3) [10]. Apart from genomic actions, TH signalling can also act by interacting with the extracellular domain of a plasma membrane protein—integrin αvβ3, which has no structural homologies with TRs. Through this extranuclear (non-genomic) mechanism, TH signalling controls the proliferation, apoptosis, the trafficking of intracellular protein, and phosphorylation/activation of TRs [11]. This finely tuned regulation of systemic and local levels of THs along with the different spatiotemporal expression of TRs and DIOs and different mechanisms of action enable TH signalling to orchestrate multiple tissue-specific functions simultaneously and to have diverse effects on animal physiology and pathobiology.

## 2. The Availability of T3 as a Determinant of Human Evolution

Millions of years ago, the first hominids had to alternate habitats periodically and were often compelled by food scarcity, violent natural phenomena, and predators to colonise new territories. These changes coincided with changes in dietary habits, encountering unfamiliar climates, physical catastrophes, and biological threats, all of which required a set of global anatomical and physiological adaptations. In our opinion, THs—which can be absorbed through the digestive system and concurrently regulate development, growth, and metabolism—must have played a pivotal role in the adaptation, survival, and thriving of early hominids.

Australopithecines were the first hominids that experienced a major ecosystem and diet shift. Apart from eating fruits, leaves, and possibly scavenging, these Homo forebears started consuming small animals, such as amphibians, birds, and reptiles. This prey was consumed whole, and the animals’ thyroid glands thus provided a significant amount of TH to their hominid predators. This shift might have initially resulted in an altered TH profile for females, which probably affected fertility negatively or may even have caused congenital anomalies [12]. This first instance of biological stress caused by high levels of exogenous TH may have served as a selection filter that favoured individuals who could tolerate sharp increases in THs. Nevertheless, the high levels of THs may also have provided an evolutionary advantage to these “more tolerant” individuals, as it could accelerate development and growth, enable the rapid regulation of the metabolism, and improve neural and possibly cognitive processes.

Homo habilis later colonised exposed habitats and became a habitual scavenger. Although these earliest representatives of our genus probably still ate some small animals and vegetation, their diet appears to have incorporated a substantial amount of marrow and brains from scavenged carcasses [13]. Such a diet would have been proportionally higher in essential fatty acids, which are indispensable for brain development and function [14]. Nevertheless, tissues and organs (including thyroid glands) are commonly consumed by the primary hunters of any prey and are not typically available to scavengers. As a consequence, Homo habilis probably assimilated less exogenous THs than Australopithecines, and this made the endogenous production of TH by their thyroid gland key to their survival. Thus, natural selection favoured those who, under these conditions of low TH intake, had the capacity to not only increase the endogenous production of THs but to also modulate the interactions of TH with cellular and nuclear receptors so as to efficiently respond to metabolic and other challenges.

An increase in externally received THs could be observed again in Homo erectus, who became a consumer of different types of animals. Once again, this shift may have contributed to the radical morphological changes in the body and brain of Homo erectus, which enabled their survival for over 1,000,000 years and made them capable of moving beyond Africa [13]. During this long journey through novel territories, Homo erectus encountered diverse stresses and challenges: hostile weather conditions (e.g., often low temperatures), long-distance walking, feasting–fasting cycles, and as a typical hunter-gatherer, frequent external traumas.

Intermittent food scarcity made it imperative to save energy by adopting an energy conservation hypothyroidism-like profile, which is typical of low activity and fasting periods [15,16]. On the other hand, a sharp drop in temperature or migration to cold habitats made effective thermoregulation a critical factor for survival. The cold stress that was experienced by Homo erectus in seasonal habitats at high latitudes and elevations would have favoured the selection of temperature-sensitive pathways that could rapidly thermoregulate themselves by modulating the basal metabolic rate (BMR). To this end, natural selection would have favoured the evolution of a TH signalling profile that could boost cellular metabolism, increase BMR, and promote thermoregulation on demand according to the environmental temperature.

Likewise, alternate periods of walking (long distances) and running along with periods of low activity (typical of the hunter/gatherer lifestyle) would have necessitated the evolution of mechanisms that could simultaneously regulate metabolism and muscle structure. In fact, the skeletal muscles are major targets of TH signalling, which governs their development, homeostasis and regeneration, contractile function, and growth in response to physical challenges [17]. As such, the capacity for the coordinated regulation of the metabolism and muscles by TH signalling could be a critical selective advantage.

The ability to maintain high levels of THs was decisive for Homo erectus’ cognitive potential, too. In fact, TH is crucial for brain development and cognitive function throughout life, from early embryogenesis to adult brain development, since it governs many aspects of neurogenesis, including proliferation, survival, cell fate decisions, migration, differentiation, growth, and the maturation of both neuronal and glial cells [18].

Individuals with TH signalling that could reactivate developmental programmes to heal wounds also had an evolutionary advantage. Through their nuclear receptors, THs can regenerate skin tissues by accelerating barrier formation and stimulating the proliferation of epidermal keratinocytes and dermal fibroblasts [19]. As such, individuals with imperfect TH signalling would have exhibited delayed wound healing (as happens in modern humans with hypothyroidism [20,21]) and may have been susceptible to wound contamination and its complications. The ability to efficiently heal wounds by modulating TH levels and TRs and DIOs expression could, in this way, be an important advantage for survival.

Modern humans (Homo sapiens) are the only surviving species of the genus Homo that evolved from their most likely recent ancestor, Homo erectus. According to the so-called “Out of Africa” theory—the dominant model of the geographic origins and early migration of anatomically modern humans—Homo sapiens first evolved in Africa and then spread around the world between 100,000 and 200,000 years ago, superseding all other hominid species. Again, the potential pressures that drove the evolution of modern humans were environmental conditions and/or changes in diet, efficacy in communicating and interacting socially, and dexterity. In addition, the eruption of a super-volcano in Sumatra around 70,000 years ago that may have led to a ‘nuclear winter’, (followed by a 1000-year ice age) would have put immense selection pressure on humans. This pressure would also have intensified the development of a TH signalling profile that could allow for greater metabolic “flexibility”, higher regenerative/repair potential, improved neuronal plasticity and advanced cognitive capacities (e.g., prediction and strategy skills), and behavioural flexibility.

**Figure 1:**
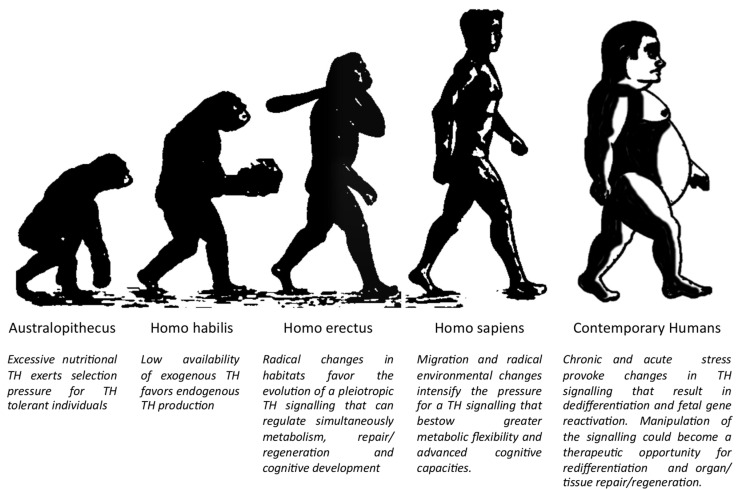
The key role of TH signalling in human evolution and disease. (Modified from Biologiwise.com accessed on 27 October 2021).

Based on the above analysis, we suggest that TH signalling evolved through millions of years as a sensitive sensor that transmitted changes from their environment to organisms and drove analogous adaptive physiological responses. This makes it a major determinant in human evolution, physiology, and disease (Figure 1).

## 3. The Fundamental Role of TH Signalling in Contemporary Modern Humans

Modern humans, despite their tremendous progress in overcoming the problems of their ancestors, face significantly diverse challenges that affect their quality of life and survival. Degradation of the environment, sedentary lifestyles, high-sugar and high-fat diets, and chronic (psychological) stress, among other factors, increase the susceptibility and predisposition of modern humans to acute injuries and chronic pathologies, respectively. For instance, the global rise in the prevalence of obesity leads, among other things, to an important increase in the prevalence of diabetes and cardiovascular diseases. Again, TH signalling seems to be highly sensible and responsive to these environmental and pathophysiological challenges.

Diabetic patients, for example, exhibit significantly lower T3 plasma levels [22,23], along with a higher prevalence of thyroid dysfunction compared to the healthy population. Moreover, hypothyroidism is the most common diabetes-associated disorder [24,25], while thyroid dysfunction and low T3 levels in diabetic patients are strongly associated with worse renal outcomes and increased mortality [26,27].

It has also long been observed that after acute injuries, such as myocardial ischaemia, there is also a drop in T3 levels [28]. Accumulative evidence suggests that the decrease in T3 is associated with increased mortality and morbidity [29]. Indeed, after acute myocardial infarction (AMI) in patients treated with mechanical revascularisation, lower free T3 levels have been associated with increased long-term mortality [29], while circulating T3 has been shown to be an independent factor that determines the recovery of the left ventricle 6 months after AMI. Furthermore, in patients with chronic heart failure, changes in T3 levels are associated both with worse functional outcomes [30,31,32] and increased cardiac mortality [33].

Although the biological significance of a decrease in TH in the aforementioned and other pathological conditions [34,35,36,37] is still under intensive investigation, it has been suggested that low T3 levels can initially provide a metabolic benefit to stressed organs. A striking example of this phenomenon is the diabetic heart, in which the decrease of T3 levels triggers a metabolic switch that is linked with the remodelling of the contractile machinery from highly energy-consuming proteins to energy-saving ones. This response may be adaptive and beneficial in the short term for stressed cardiac muscle, as it minimises catabolic phenomena and reduces energy consumption. However, in the long term, this low-energy structural and metabolic profile may be energetically inefficient and may eventually become detrimental for the tissue [9].

Based on the above clinical findings TH treatment has been used in several clinical settings for cardiovascular diseases. For instance, T3 treatment significantly increased the cardiac index of patients after coronary bypass surgery (CABG) [38], ameliorated regional dysfunction after AMI [39], improved stroke volume, and reduced the levels of the N-terminal pro b-type Natriuretic Peptide [40] in stable patients with heart failure.

Although the therapeutic mechanisms of action of TH are not well studied in humans, in vitro and in vivo disease models have provided important knowledge about the reparative and regenerative properties of TH signalling. Exposing isolated cardiomyocytes to stressful stimuli, such as adrenergic stimulation [41,42] and pro-inflammatory factors [43] have shown that the TH–TRα1 axis controls alterations in the cellular structure, size, shape, and geometry of the cardiomyocytes. In rodent models of myocardial infarction, the T3 levels decreased, and TRα1 was expressed at significantly higher levels [44], while its pharmacological inactivation was detrimental and resulted in a dramatic deterioration of heart structure and function [45]. Likewise, recent studies in our lab have shown that the glomeruli of patients and rats with diabetic nephropathy (DN) re-expressed the predominantly foetal isoform TRα1 and that these cells were also positive for a number of foetal and injury-related podocyte markers [46]. Remarkably, the simultaneous re-expression of TRα1 and foetal markers in the glomerulus has been observed in most rodent diabetes models (i.e., streptozotocin-induced diabetes, ob/ob mice and Zucker diabetic fatty rats). In rats with DN, the glomerular expression of the TH-inactivating enzyme deiodinase 3 (DIO3) increased, while the blood T3 levels were progressively decreasing and inversely correlated with the worsening of metabolic and renal disease. Furthermore, human podocytes that have been exposed to typical components of the diabetic milieu in vitro (high glucose and H_2_O_2_) exhibited markedly upregulated TRα1 and DIO3 expression. These changes in TH signalling were accompanied by cytoskeleton rearrangements, adult podocyte marker downregulation, and foetal kidney marker upregulation along with the induction of a maladaptive cell cycle, and TRα1-dependent hypertrophy.

**Figure 2:**
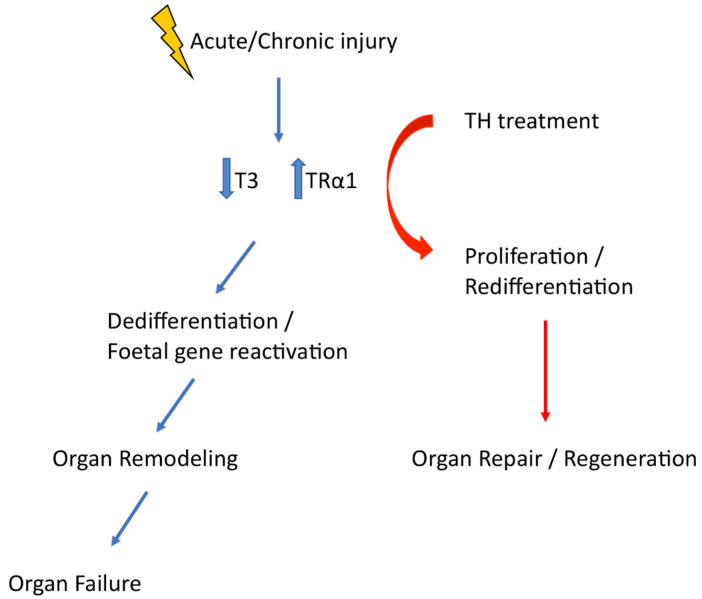
Acute of chronic injury is followed by an adoption of a foetal TH signalling profile which triggers dedifferentiation and foetal gene reactivation. Manipulation of TH signalling timely is a therapeutic opportunity that can enhance tissue repair and regeneration.

The above data suggest that the TH–TRα1 receptor axis adopts a foetal relationship profile (low T3 levels and increased TRα1 expression) that reactivates developmental programmes to drive tissue repair and regeneration (Figure 2). Although this is a regeneration imperfecta, it can be exploited therapeutically. Indeed, after myocardial infarction in rodents, in which T3 levels were found to be significantly lower after ischaemia, T3 therapy resulted in improved systolic function and geometry of the left ventricle [47], and this was associated with the activation of the pro-survival and pro-regenerative Akt signalling [48]. TH treatment also improved mechanical performance in post-infarcted diabetic myocardium, increasing cardiac mass, improving wall stress, and favourably changing the geometry of the left ventricular [49]. Finally, in the kidneys of a diabetes type 2 rodent model, TH treatment strongly reduced glomerular and tubular damage, rescued podocyte loss, significantly decreased foetal marker expression (like Pax2, Six2), and enhanced podocyte proliferation (unpublished data).

All of these data indicate that TH signalling is a key diachronic sensor and modulator of stressful stimuli in humans and, if manipulated in a timely manner, could have exciting therapeutic potential.

## 4. Discussion

The presented data and arguments that were analysed here suggest that iodotyrosines and thyroid hormones were indispensable for the emergence of life on the planet and the evolution of primitive organisms into present-day forms of life. Initially, TH served as antioxidant/catalyst and gradually evolved into a molecule that could sense environmental stimuli and regulate development, metabolism, metamorphosis and tissue repair/regeneration, and homeostasis in most living animals. In human evolution, TH signalling played a critical role in facilitating the adaptation of early hominids to persistently challenging and continuously changing environments.

Although our hypothesis is based on data analysis and speculative evidence, this does not imply that there is also an actual co-causation between the changes in TH signalling and the changes that can be observed in Homo phenotypes. For example, one could argue that TH signalling, as a highly conserved molecular pathway, could not be a major determinant of substantial intraspecific and interspecific phenotypical differences. However, some thyroid diseases indicate the opposite: single mutations in TH signalling can lead to radical phenotypical alterations and increased intraspecific variety. A striking example of this is congenital hypothyroidism—when caused by genetic mutations—which can cause profound patho-phenotypical alterations in growth, metabolism, and brain development [50].

The significance of the role of TH signalling in human evolution is also supported by the present-day differences between this signalling in modern humans and their closest relatives. Non-human apes (i.e., orangutans, gorillas, chimpanzees, and bonobo) have lower serum totals of T4 and T3 than humans [51] and exhibit significant differences in thyroid hormone metabolism [52], which may be responsible for the major variations in growth, metabolism, and development between humans and apes. For example, a small (approximately two-fold) difference in the level of a thyroid hormone-binding protein between humans and apes [52] could significantly affect brain genes and alter the trajectory and mechanisms of brain development.

Likewise, a single-nucleotide polymorphism in the DIO2 gene of Neanderthals [53] that resulted in a decreased capacity for conversion of T4 to T3 could have affected key developmental programmes and could potentially explain the significant phenotypical differences between Neanderthals and Homo sapiens despite their closeness in terms of genetic ancestry [53,54]. Most importantly, this variant may have enabled Neanderthals to hibernate and survive long and cold winters [55] (if this evidence is further confirmed), but when archaic humans switched to a high-carbohydrate diet, the variant became disadvantageous, as it is associated with an increased risk of diabetes [56,57]. These findings strongly indicate that even small changes in TH signalling might have an enormous effect on development, growth, and metabolism, and should be of utmost significance for the adaptive responses of Homo sapiens to environmental changes.

Finally, the alterations in TH signalling that are observed in modern humans suggest that these ancient evolutionary roles of TH signalling have been conserved in contemporary modern humans, in which TH signalling seems to respond and possibly contribute to adaptations to new environmental and pathogenic stresses. The pharmacological manipulation of TH in a timely manner could potentially represent a promising therapeutic strategy for repair and regeneration.
